# Supplementary material for: Impact of voluntary termination of pregnancy on female sexual function: A french monocentric longitudinal study
Source: PLoS One. 2026 Apr 15;21(4):e0346964. doi: 10.1371/journal.pone.0346964 (PMC13082641; doi:10.1371/journal.pone.0346964)
Supplement: S1 Table — Data are presented as n (%) unless otherwise indicated as median (IQR). Percentages are calculated based on the total study population corresponding to each study time point: inclusion (n = 186), 1 month (n = 80), 3 months (n = 48), and 6 months (n = 47). VTOP: Voluntary Termination of Pregnancy. P-values were calculated using Chi-square tests for independence, with a significance threshold set at 0.05. (PDF) [file pone.0346964.s001.pdf]

Characteristics	At baseline<br>n = 186	At 1 month<br>n = 80	At 3 months<br>n = 48	At 6 months<br>n = 47	p
**Age** *Median (IQR)*	*25 (22–32)*	*26 (22–32.5)*	*26 (21.5–32)*	*26 (22–33)*	0.857
18–25 years	99 (53.2)	39 (48.8)	22 (45.8)	20 (42.5)	
26–35 years	63 (33.9)	31 (38.7)	19 (39.6)	21 (44.7)	
> 35 years	24 (12.9)	10 (12.5)	7 (14.6)	6 (12.8)	
**Parity** *Median (IQR)*	*0 (0–1)*	*0 (0–1)*	*0 (0–1)*	*0 (0–1)*	0.499
No children	121 (65.1)	58 (72.5)	35 (72.9)	34 (72.3)	
At least one child	65 (34.9)	22 (27.5)	13 (27.1)	13 (27.7)	
**Number of previous VTOPs**					0.844
None	134 (72.0)	58 (72.5)	35 (72.9)	31 (66.0)	
At least one	52 (28.0)	22 (27.5)	13 (27.1)	16 (34.0)	
**VTOP method performed**					0.548
Medical	63 (33.9)	26 (32.5)	21 (43.7)	18 (38.3)	
Surgical	123 (66.1)	54 (67.5)	27 (56.3)	29 (61.7)	
**Relationship status prior to the procedure**					0.660
Single	34 (18.3)	17 (21.3)	6 (12.5)	8 (17.0)	
In a relationship	152 (81.7)	63 (78.7)	42 (87.5)	39 (83.0)	
**History of violence at least once in a lifetime**	106 (57.0)	52 (65.0)	30 (62.5)	30 (63.8)	0.451
History of sexual violence	54 (29.0)	26 (32.5)	12 (54.5)	17 (36.2)	**0.026**
History of physical violence	63 (33.9)	30 (37.5)	17 (35.4)	23 (48.9)	0.513
History of psychological violence	94 (50.5)	45 (56.3)	27 (56.3)	29 (61.7)	0.563
**Psychological symptoms before discovering the pregnancy**	158 (85.0)	62 (77.5)	40 (83.3)	36 (76.6)	0.398
Fatigue	152 (81.7)	59 (73.8)	38 (79.2)	35 (74.5)	0.446
Sadness	60 (32.3)	19 (23.8)	12 (25.0)	11 (23.4)	0.398
Anxiety	68 (36.6)	49 (61.3)	26 (54.2)	27 (57.5)	**0.002**
Guilt	36 (19.3)	13 (16.3)	11 (22.9)	9 (19.2)	0.841
**Psychological symptoms following the discovery of the pregnancy**	170 (91.4)	73 (91.3)	43 (89.6)	44 (93.6)	0.949
Fatigue	161 (86.6)	68 (85.0)	40 (83.3)	41 (87.2)	0.938
Sadness	97 (52.1)	41 (51.3)	23 (47.9)	26 (55.3)	0.924
Anxiety	88 (47.3)	43 (53.8)	22 (45.8)	22 (46.8)	0.739
Guilt	65 (35.0)	27 (33.8)	19 (39.6)	19 (40.4)	0.899
**Sexual symptoms after discovering the pregnancy**	135 (72.6)	54 (67.5)	36 (75.0)	31 (66.0)	0.648
Desire disorders	104 (55.9)	46 (57.5)	30 (62.5)	26 (55.3)	0.863
Arousal disorders	71 (38.2)	33 (41.3)	21 (43.8)	21 (44.7)	0.804
Lubrification issues	30 (16.1)	15 (18.8)	7 (14.6)	5 (10.6)	0.674
Orgasm disorders	30 (16.1)	14 (17.5)	10 (20.8)	10 (21.3)	0.789
Satisfaction issues	34 (18.3)	15 (18.8)	8 (16.7)	7 (14.9)	0.942
Dyspareunia	21 (11.3)	10 (12.5)	6 (12.5)	4 (8.5)	0.909
