# Supplementary material for: Impact of voluntary termination of pregnancy on female sexual function: A french monocentric longitudinal study
Source: PLoS One. 2026 Apr 15;21(4):e0346964. doi: 10.1371/journal.pone.0346964 (PMC13082641; doi:10.1371/journal.pone.0346964)
Supplement: S2 Table — Data are presented as n (%) unless otherwise indicated as median (IQR). VTOP: Voluntary Termination of Pregnancy. P-values were calculated using Chi-square tests for independence, with a significance threshold set at 0.05. (PDF) [file pone.0346964.s002.pdf]

Characteristics	Respondants at 6 months	No respondants at 6 months	p
	n = 47	n = 139	
**Age** *Median (IQR)*	*26 (22–33)*	*25 (22–30)*	0.172
18–25 years	20 (42.5)	79 (56.8)	
26–35 years	21 (44.7)	42 (30.2)	
> 35 years	6 (12.8)	18 (13.0)	
**Parity** *Median (IQR)*	*0 (0–1)*	*0 (0–1)*	0.301
No children	34 (72.3)	87 (62.6)	
At least one child	13 (27.7)	52 (37.4)	
**Number of previous VTOPs**			0.375
None	31 (66.0)	103 (74.1)	
At least one	16 (34.0)	36 (25.9)	
**VTOP method performed**			0.573
Medical	18 (38.3)	45 (32.4)	
Surgical	29 (61.7)	94 (67.6)	
**Relationship status prior to the procedure**			0.968
Single	8 (17.0)	26 (18.7)	
In a relationship	39 (83.0)	113 (81.3)	
**History of violence at least once in a lifetime**	30 (63.8)	76 (54.7)	0.355
History of sexual violence	17 (36.2)	37 (26.6)	0.289
History of physical violence	**23 (48.9)**	**40 (28.8)**	**0.020**
History of psychological violence	29 (61.7)	65 (46.8)	0.102
**Psychological symptoms before discovering the pregnancy**	36 (76.6)	122 (87.8)	0.106
Fatigue	35 (74.5)	117 (84.2)	0.204
Sadness	11 (23.4)	49 (35.3)	0.186
Anxiety	27 (57.5)	91 (65.5)	0.417
Guilt	9 (19.2)	27 (19.4)	1
**Psychological symptoms following the discovery of the pregnancy**	44 (93.6)	126 (90.6)	0.744
Fatigue	41 (87.2)	120 (86.3)	1
Sadness	26 (55.3)	71 (51.08)	0.738
Anxiety	22 (46.8)	76 (54.7)	0.444
Guilt	19 (40.4)	46 (33.1)	0.463
**Sexual symptoms after discovering the pregnancy**	31 (66.0)	104 (74.8)	0.323
Desire disorders	26 (55.3)	78 (56.1)	1
Arousal disorders	21 (44.7)	50 (36.0)	0.374
Lubrification issues	5 (10.6)	25 (18.0)	0.334
Orgasm disorders	10 (21.3)	20 (14.4)	0.379
Satisfaction issues	7 (14.9)	27 (19.4)	0.633
Dyspareunia	4 (8.5)	122 (87.8)	0.667
