# Supplementary material for: Impact of voluntary termination of pregnancy on female sexual function: A french monocentric longitudinal study
Source: PLoS One. 2026 Apr 15;21(4):e0346964. doi: 10.1371/journal.pone.0346964 (PMC13082641; doi:10.1371/journal.pone.0346964)
Supplement: S3 Table — Baseline variables refer to sexual activity before the discovery of the unintended pregnancy. *Percentages are calculated only among participants reporting a reduction in sexual activity or intercourse during the previous month. Data are self-reported. (PDF) [file pone.0346964.s003.pdf]

	Baseline	1 month	3 months	6 months
**Participants, n (%)**	186 (100.0)	80 (43.0)	48 (25.8)	47 (25.3)
**Sexual activity frequency in the past 4 weeks**				
≥ 3 times/week	73 (39.3)	24 (30.0)	18 (37.5)	15 (31.9)
2 times/week	45 (24.2)	13 (16.3)	9 (18.8)	12 (25.5)
1 time/week	25 (13.4)	15 (18.7)	6 (12.5)	6 (12.8)
1–3 times/month	29 (15.6)	19 (23.7)	10 (20.8)	8 (17.0)
<1/month	14 (7.5)	9 (11.3)	5 (10.4)	6 (12.8)
**Reported reduction in sexual activity during the previous month, n (%)**		46 (57.5)	25 (52.1)	23 (48.9)
**Main reported reasons for reduced sexual activity***				
Fear of a new pregnancy		14 (30.4)	10 (40.0)	6 (26.1)
Awaiting initiation of contraception		9 (19.6)	1 (4.0)	1 (4.3)
Partner-related conflicts following VTOP		2 (4.4)	2 (8.0)	3 (13.0)
None of the above		20 (43.5)	17 (68.0)	7 (30.4)
