# Supplementary material for: Impact of voluntary termination of pregnancy on female sexual function: A french monocentric longitudinal study
Source: PLoS One. 2026 Apr 15;21(4):e0346964. doi: 10.1371/journal.pone.0346964 (PMC13082641; doi:10.1371/journal.pone.0346964)
Supplement: S4 Table — Data are presented as n (%). Percentages are calculated based on the total study population at inclusion (n = 186). Sexual dysfunction is defined as an FSFI score ≤ 26.55. VTOP: Voluntary Termination of Pregnancy. P-values were calculated using Chi-square tests for independence, with a significance threshold set at 0.05. (PDF) [file pone.0346964.s004.pdf]

Characteristics	Presence of sexual dysfunction<br>n = 114	Absence of sexual dysfunction<br>n = 72	p
**Age** *Median (IQR)*			0.189
18–25 years	66 (57.9)	33 (45.8)	
26–35 years	33 (29.0)	30 (41.7)	
> 35 years	15 (13.1)	9 (12.5)	
**Parity** *Median (IQR)*			0.562
No children	76 (66.7)	45 (62.5)	
At least one child	38 (33.3)	27 (37.5)	
**Number of previous VTOPs**			0.965
None	82 (71.9)	52 (72.2)	
At least one	32 (28.1)	20 (27.8)	
**VTOP method performed**			0.163
Medical	20 (27.8)	43 (37.7)	
Surgical	52 (72.2)	71 (62.3)	
**Relationship status prior to the procedure**			0.105
Single	25 (21.9)	9 (12.5)	
In a relationship	89 (78.1)	63 (87.5)	
**History of violence at least once in a lifetime**	66 (57.9)	40 (55.6)	0.754
History of sexual violence	36 (31.6)	18 (25.0)	0.336
History of physical violence	38 (33.3)	25 (34.7)	0.845
History of psychological violence	59 (51.8)	35 (48.6)	0.676
**Self-reported psychological symptoms before discovering the pregnancy**	103 (90.4)	55 (76.4)	**0.009**
Fatigue	98 (86.0)	54 (75.0)	0.059
Sadness	45 (39.5)	15 (20.8)	**0.008**
Anxiety	44 (38.6)	24 (33.3)	0.468
Guilt	28 (24.6)	8 (11.1)	**0.024**
