# Supplementary material for: Impact of voluntary termination of pregnancy on female sexual function: A french monocentric longitudinal study
Source: PLoS One. 2026 Apr 15;21(4):e0346964. doi: 10.1371/journal.pone.0346964 (PMC13082641; doi:10.1371/journal.pone.0346964)
Supplement: S5 Table — Data are presented as n (%). Sexual dysfunction is defined as an FSFI score ≤ 26.55. VTOP: Voluntary Termination of Pregnancy. P-values were calculated using Chi-square tests for independence, with a significance threshold set at 0.05. Denominators correspond to the total population at each study time point, except for the variable “presence of sexual dysfunction,” where the number of respondents varies: For the presence of sexual dysfunction at inclusion: n = 80 at 1 month; n = 48 at 3 months; n = 47 at 6 months. For the presence of sexual dysfunction at 1 month: n = 36 at 3 months; n = 41 at 6 months. For the presence of sexual dysfunction at 3 months: n = 29 at 6 months. *: Data refer to the last month. Contraceptive methods were classified into four categories: none, barrier (condoms), natural (withdrawal or cycle tracking), and medical (pill, IUD, and implant). (PDF) [file pone.0346964.s005.pdf]

	1 months (n = 80)			3 months (n = 48)			6 months (n = 47)		
	**Sexual dysfunction** n = 39	**None** n = 41	p	**Sexual dysfunction** n = 22	**None** n = 26	p	**Sexual dysfunction** n = 17	**None** n = 30	p
**Age** *Median (IQR)*			0.554			0.338			0.420
18–25 years	20 (51,3)	19 (46.3)		9 (40.9)	13 (50.0)		9 (52.9)	11 (36.7)	
26–35 years	13 (33.3)	18 (43.9)		11 (50.0)	8 (30.8)		7 (41.2)	14 (46.7)	
> 35 years	6 (15.4)	4 (9.8)		2 (9.1)	5 (19.2)		1 (5.9)	5 (16.6)	
**Parity** *Median (IQR)*			0.716			0.978			0.634
No children	29 (74.4)	29 (70.7)		16 (72.7)	19 (73.1)		13 (76.5)	21 (70.0)	
At least one child	10 (25.6)	12 (29.3)		6 (27.3)	7 (26.9)		4 (23.5)	9 (30.0)	
**Number of previous VTOPs**			0.062			0.532			**0.015**
None	32 (82.1)	26 (63.4)		17 (77.3)	18 (69.2)		15 (88.2)	16 (53.3)	
At least one	7 (17.9)	15 (36.6)		5 (22.7)	8 (30.8)		2 (11.8)	14 (46.7)	
**VTOP method performed**			0.747			0.165			0.352
Medical	12 (30.8)	14 (34.1)		12 (54.6)	9 (34.6)		8 (47.1)	10 (33.3)	
Surgical	27 (69.2)	27 (65.9)		10 (45.4)	17 (65.4)		9 (52.9)	20 (66.7)	
**Relationship status prior to the procedure**			**0.042**			0.827			0.932
Single	12 (30.8)	5 (12.2)		3 (13.6)	3 (11.5)		3 (17.7)	5 (16.7)	
In a relationship	27 (69.2)	36 (87.8)		19 (86.4)	23 (88.5)		14 (82.3)	25 (83.3)	
**History of violence at least once in a lifetime**	24 (61.5)	28 (68.3)	0.527	14 (63.6)	16 (61.5)	0.881	10 (58.8)	20 (66.7)	0.591
History of sexual violence	13 (33.3)	13 (31.7)	0.877	12 (54.5)	8 (30.8)	0.096	7 (41.2)	10 (33.3)	0.591
History of physical violence	11 (28.2)	19 (46.3)	0.094	8 (36.4)	9 (34.6)	0.900	7 (41.2)	16 (53.3)	0.423
History of psychological violence	21 (53.8)	24 (58.5)	0.673	13 (59.1)	14 (53.8)	0.715	10 (58.8)	19 (63.3)	0.760
**Presence of sexual dysfunction at inclusion**	27 (69.2)	17 (41.5)	**0.013**	17 (77.3)	12 (46.2)	**0.028**	11 (64.7)	14 (46.7)	0.234
**Presence of sexual dysfunction at 1 month**				11 (84.6)	5 (21.7)	**<0.0001**	7 (38.9)	7 (30.4)	0.571
**Presence of sexual dysfunction at 3 months**							11 (78.6)	2 (13.3)	**<0.0001**
**Contraceptive method***			0.999			0.355			0.106
None	5 (12.8)	5 (12.2)		5 (22.7)	2 (7.7)		6 (35.3)	4 (13.3)	
Natural	1 (2.6)	1 (2.4)		1 (4.6)	3 (11.5)		0 (0.0)	5 (16.7)	
Barrier	7 (18.0)	7 (17.1)		1 (4.6)	3 (11.5)		3 (17.6)	3 (10.0)	
Medical	26 (66.7)	28 (68.3)		15 (68.1)	18 (69.3)		8 (47.1)	18 (60.0)	
**Self-reported psychological symptoms ***	35 (89.7)	33 (80.5)	0.247	21 (95.5)	16 (61.5)	**0.005**	16 (94.1)	22 (73.3)	**0.082**
Fatigue	24 (61.5)	23 (56.1)	0.621	16 (72.7)	14 (53.8)	0.178	14 (82.3)	16 (53.3)	**0.047**
Sadness	20 (51.3)	19 (466.3)	0.659	13 (59.1)	5 (19.2)	**0.004**	6 (35.3)	10 (33.3)	0.892
Anxiety	20 (51.3)	14 (34.2)	0.121	6 (27.3)	18 (69.2)	**0.004**	8 (47.1)	20 (66.7)	0.188
Guilt	16 (41.0)	9 (21.9)	0.066	7 (31.8)	3 (11.5)	0.085	5 (29.4)	2 (6.7)	**0.035**
