# Supplementary material for: Impact of voluntary termination of pregnancy on female sexual function: A french monocentric longitudinal study
Source: PLoS One. 2026 Apr 15;21(4):e0346964. doi: 10.1371/journal.pone.0346964 (PMC13082641; doi:10.1371/journal.pone.0346964)
Supplement: Attachment — Submitted filename: Response to reviewers.docx [file pone.0346964.s007.docx]

Response to Reviewers

Léa Rouchou

Department of Gynecological Surgery, Medical Gynecology, and Reproductive Medicine, Aliénor d'Aquitaine Center, Pellegrin University Hospital, Bordeaux, France.

[lea.rouchou@chu-bordeaux.fr](mailto:lea.rouchou@chu-bordeaux.fr)

13/02/2026

Editor-in-Chief

PLOS ONE

Subject: Response to Revision Requests – Manuscript [PONE-D-26-01018]

Dear Editor,

Thank you for your constructive feedback and for the opportunity to revise our manuscript. We have carefully addressed all the suggestions and requirements you mentioned and have made the necessary revisions. Below is a detailed summary of the actions we have taken in response to your comments:

1. Adherence to PLOS ONE Formatting Requirements

We have revised the manuscript to ensure it fully complies with PLOS ONE’s formatting requirements, following the provided submission templates. The document has been reformatted to meet all the required standards, and we have attached the revised version for your review.

2. Incorporation of the Title Page into the Main Document

As requested, we have incorporated the title page into the beginning of the manuscript itself, including the full list of authors and their affiliations. This title page is no longer uploaded as a separate file.

3. Third-Party Data Availability

Regarding the use of third-party data in our study, we have updated the "Data Availability Statement" to clarify that these data can be shared. We are now providing an anonymized version of the dataset in Excel format, which can be requested by other researchers.

4. Reference to Figure 2 in the Text

We have added a reference to Figure 2 in the text, as requested. The reference has been inserted at the appropriate point in the manuscript to guide readers to the figure.

5. Improved Quality of Figures 1 and 2

We have uploaded new versions of Figures 1 and 2 with enhanced resolution, as recommended.

6. Reference to Table 2 in the Text

We have added a reference to Table 2 in the text, as requested. The table is now properly cited in the manuscript to ensure readers can easily access it.

7. Captions for Supporting Information Files

We have included captions for all supporting information files at the end of the manuscript, as per PLOS ONE’s guidelines. Additionally, we have updated the in-text citations to match the new captions.

We hope these revisions meet your expectations and comply with the PLOS ONE submission guidelines. Thank you once again for your valuable feedback. Please do not hesitate to contact us if you require any further information.

Kind regards,

Dr Léa Rouchou

Dear Reviewer

We sincerely thank you for your careful reading of our manuscript and for the constructive feedback, which has helped improve the clarity, rigor, and transparency of our study. Below, we provide a detailed point-by-point response to all comments.

**Major Comments**

1. Attrition Bias and Internal Validity

*Reviewer Comment: Loss to follow-up is extreme, raising concerns about selection bias and internal validity. Recommendations include reframing conclusions, providing baseline comparisons between completers and non-completers, and performing sensitivity analyses.*

Author Response:

We have explicitly reframed our conclusions throughout the manuscript, including the abstract, to clarify that results are conditional on follow-up completion.

A baseline comparison between completers and non-completers at 6 months has been added (S2 Table). No significant differences were found, except for a slightly higher prevalence of reported physical violence among respondents (p = 0.020).

A conservative sensitivity analysis was conducted assuming that participants with missing follow-up FSFI data did not change their sexual function status. Logistic mixed-effects modeling, adjusting for age, parity, relationship status, history of violence, and self-reported psychological symptoms, confirmed a significant reduction in sexual dysfunction over time (adjusted OR 0.84; 95% CI 0.75–0.95, p = 0.007).

These analyses are now described in the Methods and Results sections.

2. Absence of a True Pre-Pregnancy Baseline

*Reviewer Comment: Baseline FSFI measurement occurred after pregnancy discovery, complicating causal interpretation. Recommendations include clearer language distinguishing effects of pregnancy, VTOP, and natural temporal regression.*

Author Response:

We have revised the manuscript to consistently use observational language (e.g., “after pregnancy resolution” instead of implying post-VTOP recovery).

The Discussion now more explicitly distinguishes between the effects of unintended pregnancy, the resolution of pregnancy, and the potential influence of VTOP.

3. Sample Size Justification and Statistical Power

*Reviewer Comment: Sample size was empirically determined, limiting confidence in null findings.*

Author Response:

We clarified that several analyses are exploratory and have revised the text to avoid overinterpretation of non-significant associations, particularly for secondary outcomes (e.g., history of violence, VTOP method).

4. Measurement of Psychological Covariates

*Reviewer Comment: Psychological symptoms were assessed with non-validated items, limiting reproducibility.*

Author Response:

Terminology throughout the manuscript has been updated to “self-reported psychological symptoms” to reflect the non-validated nature of these measures.

We have acknowledged this limitation explicitly in the Discussion and avoided clinical labeling where diagnostic thresholds were not applied.

5. Generalizability

*Reviewer Comment: Monocentric design and specific participant characteristics limit generalizability.*

Author Response:

We expanded the Discussion to explicitly note limitations in generalizing results to medical abortion settings, women with psychiatric comorbidities, and populations with limited digital access.

6. FSFI Questionnaire Validation

*Reviewer Comment:* *Methods should justify use of FSFI cutpoint (≤26.55) and its applicability to the French population.*

Author Response:

The Methods now describe the FSFI as a validated 19-item tool in French, assessing six sexual function domains.

The rationale for using the ≤26.55 cutpoint as a binary outcome (sexual dysfunction vs. no dysfunction) is clarified, including its validation and alignment with clinical practice guidelines.

7. Discussion Enrichment

*Reviewer Comment: Discuss how reliance on FSFI cutpoint and self-report affects interpretation of prevalence, longitudinal change, and clinical relevance.*

Author Response:

The Discussion now emphasizes the absence of a true pre-pregnancy baseline and the potential influence of pregnancy-related stress on observed improvements.

We noted that FSFI scores are validated as categorical diagnostic thresholds rather than continuous change scores; therefore, longitudinal analyses focus on prevalence changes.

Observational language has been used to avoid causal overinterpretation.

**Minor Comments**

1. FSFI Interpretation

*Reviewer Comment: Clarify whether domain-level changes exceed minimal clinically important differences.*
Author Response: Updated in Results and Discussion to provide these details.

2. Figures

*Reviewer Comment: Figure 2 should display raw numerators (n/N) alongside percentages.*

Author Response: Figure 2 has been updated accordingly.

3. Language

*Reviewer Comment: Revise causal statements in the Discussion.*

Author Response: All statements implying causality have been revised to reflect observational study design.

We sincerely thank you again for your thoughtful comments. All suggestions have been addressed to improve transparency, methodological clarity, and interpretability.

Kind regards,

Dr Léa Rouchou
